# Climate change adaptation and mitigation in different livestock production systems and agro-ecological zones in South Africa: A systematic review

**DOI:** 10.1007/s11250-025-04660-9

**Published:** 2025-10-15

**Authors:** Mashford Zenda

**Affiliations:** Centre for Ecological Intelligence, Faculty of Engineering and the Build Environment (FEBE), Electrical and Electronic Engineering Science, University of Johannesburg, Auckland Park Campus, Auckland Park, Johannesburg, South Africa

**Keywords:** Farm level, Innovative approaches, Resilience, Strategy adoption, Sustainability

## Abstract

Livestock production in South Africa faces numerous challenges due to climate change, resource limitations, and economic constraints. Climate change adaptation and mitigation strategies are essential to ensure sustainability. This systematic literature review explores the adaptation and mitigation strategies employed in livestock production systems in South Africa. The literature review used a systematic approach to identify relevant studies using Google scholar, Scopus and Web of science. To ensure the relevance and quality of the selected studies, specific inclusion and exclusion criteria were applied. Studies were included if they addressed adaptation and or mitigation strategies in livestock production, were specific to the South African context, and were published between 2000 and 2023. Conversely, studies were excluded if they focused on regions outside South Africa, did not specifically examine livestock adaptation or mitigation, or lacked methodological rigor. This approach allowed the author to identify and synthesise a wide range of literature on the topic. Based on the inclusion criteria for the literature review, an initial screening of 330 articles was conducted, resulting in 55 articles meeting the criteria and included in the systematic review. This rigorous process helped to identify the high-quality and relevant studies on the topic. The data extracted from the 55 articles were then analysed and synthesised to identify adaptation and mitigation strategies of livestock production systems in South Africa. This helped to identify similarities and differences within the literature and supported drawing conclusions about adaptation and mitigation strategies in South African livestock production systems. Key practices include destocking during dry months, selective breeding, water resource management, construction of shade to reduce heat, financial planning, feed supplementation, and innovative approaches like wildlife ranching. These strategies, when adopted at farm level enhance resilience, productivity, and environmental conservation. Demographic, environmental, socioeconomic, and knowledge-related factors influence strategy adoption. Research progress shows increasing interest and diverse methodological approaches, indicating a growing awareness of livestock production resilience. Collaborative efforts are crucial for advancing sustainable practices and maintaining the sector’s long-term sustainability.

## Introduction

The livestock industry supports the livelihoods of approximately one billion of the most economically disadvantaged individuals globally and provides employment for nearly 1.1 billion people (Singh and Singh 2023). In this study, the term ‘livestock’ refers specifically to ruminants namely cattle, sheep, and goats which are central to rural livelihoods across much of South Africa. Livestock industry is critical for global food security and economic development, yet it also significantly contributes to greenhouse gas emissions, primarily methane (CH_4_) and nitrous oxide (N_2_O) (Truong et al. 2018; Musa 2020; Chang et al. 2021). Climate change directly impacts livestock, affecting growth rates, reproductive performance, morbidity, mortality, and feed availability (Cheng et al. 2022; Davis 2023). To address these challenges, farmers across different regions have adopted diverse strategies to adapt to climate change. For example, in Ethiopia, climate-informed farming practices reduced animal numbers are observed (Belay et al. 2023). Livestock farmers in Benin’s dry and sub-humid tropical zones employ mobility, integration with crop husbandry, and concentrate feed usage as adaptation strategies (Idrissou et al. 2020). Indigenous Fulani herders also use mobility, feed diversification, labor division, and stress management (Lorusso et al. 2013; Idrissou et al. 2023).

In South Africa, the implementation of climate change adaptation and mitigation strategies in livestock production has become increasingly vital, given the growing impacts of climate variability on rural livelihoods and food security. Climate-Smart Agriculture (CSA) stands out as a key strategy for smallholder livestock farmers seeking to boost productivity sustainably while addressing climate-related challenges. Smallholder livestock farmers, in particular, are showing increased engagement with CSA approaches, reflecting a heightened awareness of the link between climate resilience and livestock systems (Molieleng et al. 2021). These approaches often include measures such as complementary breeding of livestock (Scholtz et al. 2013; Hawkins et al. 2022; Slayi et al. 2022) and the early sale of livestock (Maluleke and Mokwena 2017; Bahta 2020). Together, these actions strengthen farmers capacity to adapt to climatic pressures.

Smallholder livestock farmers, often operating within communal settings, play a crucial role in the rural economy of South Africa. They contribute significantly to employment, supporting up to 70% of the rural population in certain areas (Nyam et al. 2020). These farmers are responsible for about 45% of the country’s agricultural production, underlining their importance to both food security and economic resilience (Nyam et al. 2020; Nwafor and Nwafor 2020). Typically, they raise indigenous livestock breeds that are well-suited to local conditions and fulfill various functions, including providing food, generating income, and acting as a buffer against food insecurity (Malusi et al. 2021).

Communal livestock farming is mainly practiced in rural regions, where smallholder farmers rely on traditional methods. This sector plays a vital economic role for many households, offering not just meat, but also essential resources like milk, draught power, and manure for crop cultivation (Cheteni and Mokhele 2019; Myeki and Bahta 2021; Zhou et al. 2022). Typically, the communal farming system operates through informal markets and meets subsistence needs. Livestock are usually grazed on natural pastures, requiring fewer inputs compared to more intensive commercial farming systems (Molotsi et al. 2017; Malusi et al. 2021). In contrast, commercial livestock farming in South Africa is marked by greater efficiency and higher levels of productivity. This approach is mainly adopted by commercially-focused farmers who manage larger herds and apply modern techniques such as feedlot management and rotational grazing (Molotsi et al. 2017; Malusi et al. 2021; Molieleng et al. 2021).

South Africa’s livestock production plays a significant role in addressing household food insecurity and poverty. However, the agriculture sector faces challenges such as climate change impacts leading to issues like high livestock death rates, slow growth, and reduced milk production (Maluleke et al. 2020). Mitigation strategies to address climate change by smallholder livestock farmers in South Africa are crucial for ensuring the sustainability of agricultural practices in the face of changing environmental conditions. Mitigation strategies encompass a range of approaches aimed at reducing greenhouse gas emissions and minimising human contributions to climate change (Rust and Rust 2013; Sarma et al. 2024). Smallholder livestock farmers in South Africa are at risk from climate change because their livelihoods depend heavily on agriculture.

Climate change significantly threatens multiple regions in South Africa, especially arid and semi-arid areas (Zenda 2024). These zones feature delicate ecosystems that are highly vulnerable to extreme weather events like droughts and heatwaves, conditions that are worsened by climate change. The consequences of these vulnerabilities go beyond environmental issues, affecting socioeconomic aspects such as food security and the stability of local economies (Patrick 2021; Mthembu and Hlophe 2021). The adoption of effective adaptation and mitigation strategies that take local contexts into account has been insufficient. Accordingly, targeted adaptation and mitigation strategies tailored to these distinct regional vulnerabilities are essential for enhancing resilience and sustainable development.

In South Africa, various national and regional bodies are key players in adapting to and mitigating climate change impacts within the livestock sector. However, the fragmented structure of these institutions hinders unified and efficient responses, as highlighted by Akanbi et al. (2021). The government is progressing with legislative initiatives like the Climate Change Bill and the National Adaptation Plan, but there are concerns that current laws fall short in fully addressing the intricate challenges climate change poses to agriculture and livestock management (Akanbi et al. 2021). At the core of South Africa’s institutional support framework is the Department of Environment, Forestry and Fisheries (DEFF), which is tasked with formulating policies and regulations that address climate change across various sectors, including agriculture and livestock (Akanbi et al. 2021). Furthermore, the National Disaster Risk Management Policy Framework highlights the importance of effective disaster risk management in the face of climate variability, with particular emphasis on livestock systems (Orievulu and Iwuji 2021). This framework advocates for proactive strategies to mitigate the impacts of droughts and other climate-related hazards that significantly affect the agricultural sector.

A study has shown that smallholder farmers are aware of the effects of climate change and are adopting various adaptation strategies to mitigate its negative impacts (Myeni and Moeletsi 2020). These strategies include changes in local practices, structural measures such as government relief programs, and technical interventions like rain harvesting (Ruwanza et al. 2022). Additionally, communal feedlots have been identified as a sustainable livelihood option that enhances livestock productivity and fosters economic opportunities for smallholder farmers, contributing to climate change resilience (Slayi et al. 2024). The other mitigation efforts in livestock production include improved livestock management practices, diet efficiency, genetic management and manure management (Moeletsi and Tongwane 2015; Bahta 2021).

Adoption of climate-smart livestock management options, particularly cost-saving measures for smallholder farmers, has been observed in Zimbabwe (Phiri et al. 2021; Muzorewa 2021; Magwegwe et al. 2024) Additionally, diversification of crops and livestock, is being explored in South Africa (Zwane 2019, Maluleke et al. 2020; Lottering et al. 2021). Despite these efforts, systemic challenges remain such as securing policy support, enhancing farmer awareness and integrating indigenous knowledge but they also offer opportunities for innovation, education, and policy development to strengthen climate adaptation and mitigation in livestock production (Singh and Singh 2023).

This systematic review aims to comprehensively examine and evaluate the various adaptation and mitigation strategies implemented in livestock production sector of South Africa. By synthesising existing literature and studies on this topic, the review seeks to provide insights into the effectiveness, challenges, opportunities and gaps in current strategies. Ultimately, the goal is to inform future research directions, policy interventions, and practical actions to enhance the resilience, sustainability and productivity of livestock in South Africa amidst climate change challenges.

## Literature search and selection

**Fig. 1:**
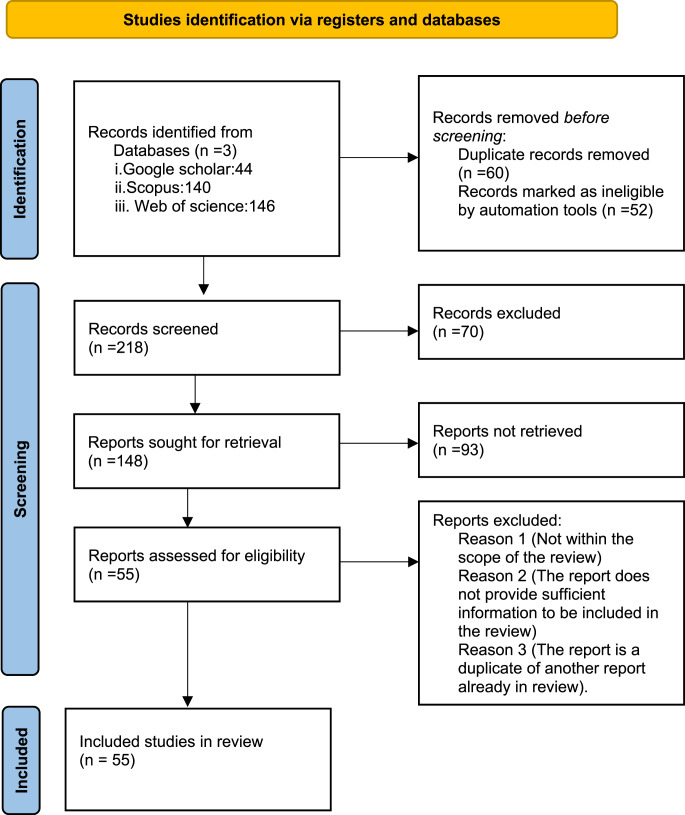
Prisma flow diagram for the current study

The literature search for this systematic review focused on articles published from 2000 onwards, targeting relevant studies on adaptation and mitigation strategies in livestock production within South Africa (Fig. 1). Google Scholar, Scopus, and Web of Science were selected as the primary databases due to their broad interdisciplinary coverage and comprehensive indexing of peer-reviewed literature relevant to adaptation and mitigation strategies in livestock production.

The initial search employed a combination of keywords including *“livestock production,” “adaptation strategies,” “mitigation strategies,” “South Africa,” “climate change,” “greenhouse gas emissions,”* and *“sustainable agriculture.”* This manual search yielded a substantial number of results, which were then filtered based on the following inclusion and exclusion criteria:

### Inclusion criteria

Studies were included if they:

- Addressed adaptation and/or mitigation strategies in livestock production.
- Were specific to the South African context.
- Were published between 2000 and 2023.

### Exclusion criteria

Studies were excluded if they:

- Focused on other regions outside South Africa.
- Did not specifically examine livestock adaptation or mitigation.
- Lacked methodological rigor.

Abstracts and full texts of potential articles were thoroughly reviewed to ensure relevance and alignment with the research focus. Articles that did not provide substantial insights into adaptation and mitigation strategies in South African livestock production were excluded during this phase. To ensure quality and reliability, preference was given to peer-reviewed journal articles, academic publications, and reputable conference proceedings. Non-peer-reviewed sources and studies lacking sufficient methodological rigor were excluded from the final selection.

Three independent reviewers conducted the screening of titles, abstracts, and full texts. Discrepancies in selection were resolved through discussion or adjudicated by a third reviewer when consensus could not be reached. This approach was adopted to maintain the objectivity and reliability expected of a systematic review.

Data was systematically extracted on study characteristics, methodological approaches, and key findings, with procedures in place to avoid distortion or bias. The quality of included studies was assessed using the AMSTAR 2 checklist, which evaluated elements such as literature search adequacy and transparency of reporting, and the Cochrane Risk of Bias Tool to identify selection, performance, and reporting biases. To synthesize the evidence, a combination of narrative synthesis, meta-analysis, and thematic analysis was applied. Narrative synthesis facilitated clustering of results and identification of gaps in the literature; meta-analysis was conducted where methodological homogeneity allowed, involving recalculation of effect sizes and confidence intervals; and thematic analysis explored overarching themes and implications for future research and practice. This multi-layered methodological approach ensured rigorous evaluation and integration of findings to enhance the credibility and utility of the review’s conclusions.

## Information retrieval and analysis

The synthesis was methodologically robust, incorporating a diverse array of research methodologies, namely quantitative, qualitative and mixed-method approaches. This comprehensive strategy enabled a nuanced exploration of the research problem from multiple perspectives, facilitating a deeper understanding of the phenomenon under investigation.

Furthermore, the integration of mixed-method approaches allowed for the triangulation of findings, validating results across different methodological lenses. By combining quantitative rigor with qualitative depth, the synthesis achieved a more comprehensive and holistic understanding of the research topic, enhancing the credibility and validity of the conclusions drawn.

## Results

### Current research trends in livestock adaptation and mitigation strategies in South Africa

Livestock production in South Africa plays a significant role in the country agricultural sector, contributing substantially to food security (Meissner et al. 2014). However, the sector is facing challenges due to climate change, including droughts and extreme heat waves, which pose threats to agricultural development and food security (Oduniyi et al. 2020a). In addition, the sustainability of livestock farming systems in South Africa is jeopardised by climate-related events and poor adaptive capacity (Oduniyi et al. 2020b). Research has identified climate change risks to communal livestock production in South Africa, emphasising limitations in research, such as the underrepresentation of different provinces within the country (Zhou et al. 2022). According to the current research progress on adaptation and mitigation strategies of livestock production in South Africa has seen significant development across various provinces and years.

**Fig. 2:**
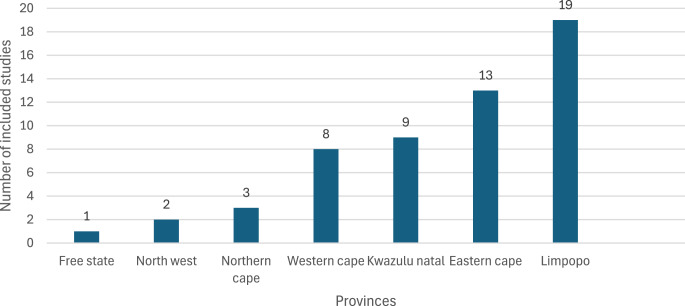
Number of included studies per province

A total of 55 studies have been conducted, with notable concentrations in certain provinces (Fig. 2). The graph illustrates the distribution of studies across various provinces, showing a clear upward trend from left to right. The Free State has the fewest studies, with only one. This number slightly increases in the North West province with two studies, followed by the Northern Cape with three. The upward trend continues with the Western Cape, which has eight studies. KwaZulu-Natal slightly surpasses the Western Cape with nine studies. A more noticeable increase is observed in the Eastern Cape, where the number of studies rises to thirteen. The trend peaks in Limpopo, which has the highest count of nineteen studies, indicating a strong focus or interest in this province compared to others. Overall, the graph depicts a steady increase in the number of studies from the Free State to Limpopo province.

The spatial distribution of research on adaptation and mitigation strategies for livestock production across South African provinces highlights important regional engagement and focus areas. The concentration of studies in certain regions likely reflects the region’s susceptibility to climate change. Livestock production varies across provinces in South Africa, with the Limpopo province expected to be the most impacted by climate change, potentially leading to changes in land use by livestock farmers (Otieno et al. 2023). The Limpopo province stands out with nineteen studies, possibly due to the impact of climate change on the livestock systems in the region (Mpandeli et al. 2015; Tshikolomo et al. 2022). The high number of studies focused on the Limpopo province highlights the need for targeted support and interventions to help livestock farmers in this region build resilience to climate change. By understanding the specific challenges and opportunities in this province, researchers, policymakers, and practitioners can work together to develop effective strategies for mitigating the impacts of climate change on livestock production and ensuring a sustainable future for farmers in the Limpopo province.

Between 2000 and 2023, the Eastern Cape province of South Africa experienced a notable increase in climate change studies, highlighting the growing recognition of the region’s vulnerability to climate-related impacts (Goqwana et al. 2008; Taruvinga et al. 2013; Popoola et al. 2019; Diniso et al. 2022; Slayi et al. 2023a). This surge in research is driven by the pressing need to understand and adapt to the adverse effects of climate change on agriculture, water resources, and overall livelihoods in the area. Recent studies have highlighted the significant challenges posed by climate change in the Eastern Cape, particularly regarding agricultural productivity. For instance, Slayi et al. (2023a) emphasises that farmers in the region are increasingly facing adverse effects such as changing rainfall patterns and prolonged droughts, which have led to livestock deaths and threaten traditional farming practices. Similarly, research by Diniso et al. (2022) indicates that dairy farmers are particularly vulnerable, necessitating adaptations such as the construction of protective structures for livestock to mitigate heat stress. These findings underscore the urgency of developing localized adaptation strategies to enhance resilience among farming communities.

According to climate projections, the Western Cape province can expect increased warming, drying, and more extreme weather events (Quintana et al. 2023). These projected changes underscore the urgent need for adaptive measures, resilience-building strategies, and proactive planning to mitigate the impacts of climate change in the Western Cape province (He and Ding 2021). By focusing on adaptive practices, building resilience, and leveraging supportive policies, the province can better manage the impacts of climate change and ensure the sustainability of its livestock production systems.

The climate change studies within the livestock sector in KwaZulu-Natal Province are evident through various research efforts that highlight the significant impacts of climate variability on livestock farming. The region has been particularly vulnerable to climate change, with studies documenting adverse effects such as drought and changing rainfall patterns that directly influence livestock production and farmer livelihoods (Mthembu and Zwane 2017; Lottering et al. 2021; Maziya 2022). The adoption of mitigation and adaptation strategies is becoming increasingly critical to ensure the long-term sustainability of the livestock sector in the province.

The impact of climate change, drought, and the need for adaptive capacity among smallholder livestock farmers in Limpopo has been a subject of research (Mpandeli et al. 2015; Tshikolomo et al. 2022). In contrast, the Northern cape province has been affected by recurring droughts, which have had unpredictable impacts on livestock production over time (Bahta and Myeki 2022). As droughts become more frequent and severe, smallholder farmers in both provinces face significant challenges in maintaining livestock productivity, accessing water and ensuring their livelihoods (Tshikolomo et al. 2022). Building adaptive capacity is crucial to enable these farmers to respond effectively to climate-related stresses, reduce their vulnerability, and promote resilience in the face of uncertainty.

The presence of studies in other provinces like Northern Cape, North West and Free state province indicates valuable insights into environmental pressures and potential adaptation strategies (Botai et al. 2016, Oduniyi et al. 2020b; Danso-Abbeam et al. 2021; Letsoalo et al. 2023). These studies demonstrate the need for a comprehensive understanding of climate change impacts and adaptation strategies across various regions, allowing for more effective and targeted interventions to support vulnerable communities.

Overall, the available research on climate change adaptation and mitigation strategies for livestock production in South Africa highlights regional variations in focus, with some provinces, like Limpopo and Eastern Cape, receiving greater attention due to their heightened vulnerability (Zhou et al. 2022). However, this uneven representation reveals a significant gap in research across other provinces, such as the Northern Cape, North West and Free State, where studies remain limited despite the challenges posed by climate change, including recurring droughts and environmental pressures. This imbalance in research efforts underscores the need for a more comprehensive and inclusive approach, ensuring that all regions, particularly underrepresented areas, are equipped with localised adaptation strategies to bolster resilience. By expanding research to include these less-studied provinces, policymakers and stakeholders can develop targeted interventions that address the diverse and region-specific challenges faced by livestock farmers across South Africa.

**Fig. 3:**
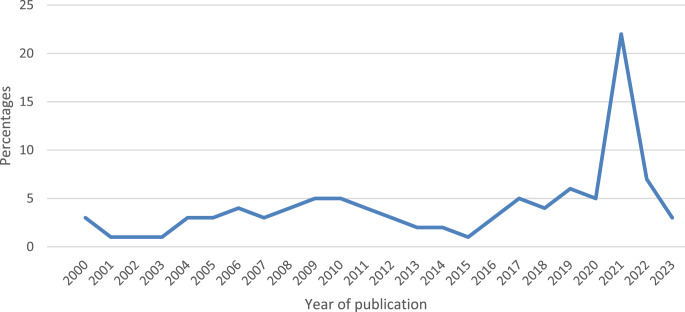
Percentage of trends in included studies per year

The graph in Fig. 3 illustrates the trends in the percentage of included studies per year from 2000 to 2023. From 2000 to 2010, the percentage remains consistently low, fluctuating between 0 and 5%, indicating a stable trend with minimal increases. Between 2011 and 2016, there’s a slight rise in the percentage of included studies, with noticeable peaks around 2013 and 2016, though the overall trend remains below 5%. A significant peak occurs between 2020 and 2021, with the percentage sharply rising to its highest point in 2021 at over 20%, reflecting a substantial increase in included studies during these years. After the peak in 2021, a noticeable decline is observed in 2022 and 2023, with the percentage decreasing, yet not returning to the lower levels seen in the early 2000s.

The surge in research activities targeting adaptation and mitigation strategies within South African livestock production, in 2021 underscores a critical response to the evolving challenges faced by the agricultural sector as a result of climate change. This heightened focus can be attributed to several interconnected factors. Firstly, the impact of climate change has become increasingly pronounced, with South Africa experiencing shifts in temperature patterns, altered precipitation regimes and more frequent extreme weather events. These changes directly affect livestock farming, posing challenges such as heat stress, water scarcity and a decrease in forage availability. These challenges are particularly pronounced in arid and semi-arid areas where more frequent droughts are reducing vegetation cover and livestock populations (Zhou et al. 2022).

Furthermore, agricultural drought is emerging as a major stressor on livestock production in the temperate and humid regions of South Africa (Myeki and Bahta 2021). Adaptation strategies by farmers in South Africa seem to be failing to effectively mitigate the impacts of climate change, as evidenced by annual livestock losses. Furthermore, the changing environment in South Africa, characterised by more extreme temperatures and increasingly variable precipitation, is expected to become more commonplace, with Sub-Saharan Africa being particularly at risk due to already high average temperatures and frequent droughts (Conradie et al. 2019). The impact of climate change on livestock farming in South Africa is multifaceted, affecting various aspects of production and sustainability. It is crucial for farmers and policymakers to implement effective adaptation strategies to mitigate these challenges and ensure the resilience of the livestock farming sector in the face of ongoing climate change.

Secondly, the recognition of agriculture’s significant contribution to greenhouse gas emissions has prompted a reevaluation of practices within the livestock sector. As global efforts to combat climate change intensify, there’s a growing imperative to reduce emissions associated with livestock farming, particularly methane produced by ruminants. The environmental efficiency of livestock production systems is a key concern, with a focus on adopting management practices that mitigate greenhouse gas emissions while maintaining productivity levels (Smith 2023). Innovative approaches such as closed-loop waste valorisation paradigms are being developed to tackle challenges associated with manure production and ammonia emissions from intensive livestock farming. The adoption of specific tools for assessing agricultural biodiversity in organic livestock farms underscores the importance of informed policies and practices to promote sustainability.

Thirdly, advancements in technology and scientific understanding have opened up new avenues for innovative solutions in livestock production. From precision farming techniques to genetic selection for climate resilience, researchers are exploring diverse strategies to enhance the adaptive capacity of livestock systems while minimizing their environmental footprint (Sharma et al. 2024). Genomic data play a vital role in managing genetic resources and improving climate resilience in livestock, as seen in studies on local adaptation and climate resilience in sheep (Tsartsianidou et al. 2021). Selective breeding for resilience traits is highlighted as a strategy to mitigate the effects of climate change on livestock production (Sánchez-Molano et al. 2019, 2020).

Moreover, the application of precision livestock farming (PLF) technologies, such as IoT-based solutions, is transforming livestock management by enabling real-time monitoring of animal health and environmental conditions (Caria et al. 2019; Mate et al. 2022). These technologies not only improve productivity but also contribute to sustainable farming practices by reducing environmental degradation and enhancing animal welfare (Mate et al. 2022). Additionally, the integration of blockchain technology in livestock management systems aims to provide intelligent solutions for farmers to meet the increasing demand for livestock products while addressing concerns about sustainability and animal welfare (Schillings et al. 2021).

Furthermore, the socioeconomic context of South Africa, with its diverse agricultural landscape and substantial reliance on livestock for livelihoods and food security, amplifies the urgency of addressing these challenges. Sustainable and resilient livestock production not only contributes to economic stability but also supports rural communities and fosters inclusive growth (Zenda and Malan 2021).

In response to these drivers, research efforts have intensified across various fronts. Studies are investigating the genetic traits associated with heat tolerance and drought resistance in livestock breeds endemic to South Africa (Molotsi et al. 2017; Van Marle-Köster et al. 2021). The genetic evaluation of indigenous livestock breeds is crucial, as these breeds have evolved under harsh environmental conditions and possess unique adaptive traits that can be leveraged for improved resilience to climate stressors (Houaga et al. 2023). A review of drought impacts and responses among smallholder farmers in South Africa highlights the critical need to understand genetic traits that can mitigate drought-related challenges in livestock (Ruwanza et al. 2022). Innovations in feed formulations aim to improve nutrient efficiency and reduce methane emissions from enteric fermentation. Additionally, there’s a growing emphasis on holistic management approaches that integrate livestock farming with ecosystem conservation and climate adaptation strategies.

Collaboration should be fostered between researchers from various fields, including agriculture, environmental science, and economics, to enrich the discussion on sustainable livestock production practices. By harnessing multidisciplinary expertise and embracing innovation, South Africa can foster a more sustainable, resilient, and climate-smart livestock production system that can thrive amidst ongoing environmental and socioeconomic challenges.

**Fig. 4:**
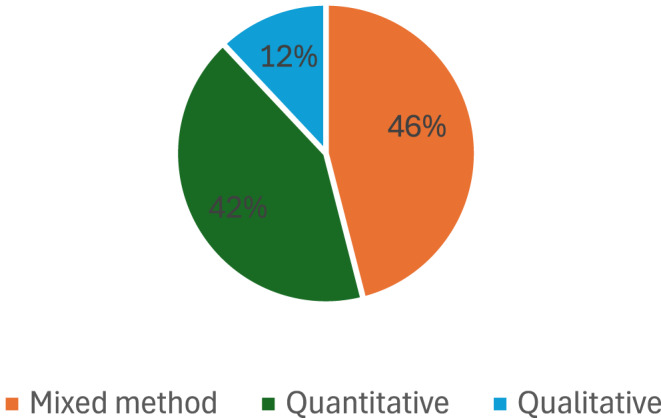
Percentage of methodology in the included study

The methodological approach employed in the research on climate change adaptation and mitigation strategies in South African livestock production is diverse and comprehensive. Out of the 55 studies conducted, 46% of the studies utilised mixed methods, combining quantitative data analysis with qualitative insights (Fig. 4). This mixed approach provides a deeper insight into the complex dynamics and factors shaping adaptation and mitigation strategies. Additionally, 42% of the studies focused solely on quantitative methods, indicating a strong emphasis on statistical analysis and numerical data in understanding livestock production systems. On the other hand, 12% of the studies relied solely on qualitative methods, emphasising in-depth interviews, observations, and textual analysis to gather insights into the topic. The combination of mixed methods, quantitative approaches, and qualitative methodologies contributes to a holistic and thorough examination of adaptation and mitigation strategies in South African livestock production.

In the context of South African livestock production, which faces numerous challenges related to climate change, resource constraints, and socio-economic dynamics, a holistic approach that combines mixed methods, quantitative analysis, and qualitative research is essential. Such an approach not only enables researchers to identify effective adaptation and mitigation strategies but also facilitates the development of policies and interventions that are informed by a deep understanding of the local context and stakeholders needs. Ultimately, by integrating diverse research methodologies, a more robust and actionable knowledge base can be generated to support sustainable livestock production in South Africa.

The provincial distribution of studies reveals a diverse agricultural landscape and varying challenges across different regions. Apart from the Limpopo, Eastern Cape, Western Cape and KwaZulu Natal provinces, other regions like Free State, North West and Northern Cape have also been studied, albeit to a lesser extent. The research progress signifies a concerted effort to advance knowledge and practices related to livestock production resilience in South Africa, taking into account the unique challenges and opportunities present in different provinces and utilising diverse methodological approaches to gain comprehensive insights.

### Adaptation and mitigation strategies of livestock production in South Africa

The current study reviewed 55 publications that reported various adaptation strategies to address the impacts of climate change on livestock production (Table 1). Adaptation and mitigation strategies are crucial components of sustainable livestock production in South Africa, especially in the face of climate change, resource limitations, and economic challenges. These strategies, observed across various studies, encompass a range of practices aimed at enhancing resilience, improving resource management, and ensuring the long-term viability of livestock farming. One key strategy observed in South Africa is destocking during dry months, a practice that involves reducing livestock numbers to align with available forage and water resources (Goqwana et al. 2008; Rasch et al. 2016; Popoola et al. 2019; Ojo et al. 2021; Zhou et al. 2022). This practice involves reducing livestock numbers to ensure sustainability and resilience in the face of environmental challenges (Vignal et al. 2023). This approach prevents overgrazing, maintains pasture health and reduces the risk of resource depletion during periods of scarcity. Concurrently, farmers often seek veterinary advice and extension services to manage livestock health effectively, implement sustainable practices, and adopt technologies that enhance productivity and resilience.

**Table 1:** Adaptation and mitigation strategies adopted by livestock farmers

Adaptation and mitigation strategies	Authors
Destocking during dry months and seeking veterinary advice and extension services.	Goqwana et al. (2008); Rasch et al. (2016); Popoola et al. (2019); Ojo et al. (2021); Zhou et al. (2022).
Complementary breeding of livestock.	Dube and Jury (2000); Below et al. (2010); Mandleni and Anim (2011a); Rust and Rust (2013); Scholtz et al. (2013); Mpandeli et al. (2015); Mare et al. (2018); Nyoni et al. (2019); Popoola et al. (2019); Zwane (2019); Malusi et al. (2021); Halimani et al. (2021); Hawkins et al. (2022); Slayi et al. (2022).
Drilling boreholes, tree planting and shade construction.	Maponya and Mpandeli (2013); Katiyatiya et al. (2017); Mare et al. (2018); Zwane (2019); Oduniyi et al. (2020a); Pili (2020); Zhou et al. (2022).
Reducing production, seeking assistance, obtaining credit, migrating.	Below et al. (2010); Zwane (2019); Bahta (2020); Fanadzo (2021); Hunter and Cronin (2020); Bahta and Myeki (2021); Bahta (2022).
Selling livestock early.	Boone et al. (2004); Mandleni and Anim (2011b); Maluleke and Mokwena (2017); Bahta (2020).
Registering as livestock farmers.	Maluleke and Mokwena (2017); Bahta (2022); Bahta and Myeki (2022)
Utilising savings and investments.	Bahta (2022); Bahta and Myeki (2021)
Supplementing livestock feed.	Gbetibouo et al. (2010); Mandleni and Anim (2011b); Lamega et al. (2021); Malusi et al. (2021); Zhou et al. (2022); Letsoalo et al. (2023).
Alternative breeding objectives.	Molieleng et al. (2021); Bahta (2022); Bahta and Myeki (2022).
Wildlife Ranching.	Otieno et al. (2023).
Crop Residues.	Lamega et al. (2021).
Changing area of land grazed.	Gbetibouo et al. (2010); Popoola et al. (2018); Hunter and Cronin (2020).
Controlled feeding, livestock migration, quarantine, buying feed.	Fanadzo (2021).
Winter Feeding.	Goqwana et al. (2008).
Annual flush of vegetation.	Hahn et al. (2005).
Genetic Diversity.	Molotsi et al. (2017).
Dip and Dose, Exchange stock, Fence camps, Portable water.	Mandleni and Anim (2011b).
Use of Indigenous plant to boost production.	Kunene et al. (2003).
Continuous grazing under conservative stocking rates.	Rasch et al. (2016); Malusi et al. (2021).
Wetting and forced ventilation.	Ogundeji et al. (2021).
Seeking shade, extending their limbs, and wallowing in mud, reducing food intake.	Preez (2007).
Acaricides every fortnight and consider hair length and coat color when acquiring cattle.	Katiyatiya et al. (2014).
Changing grazing routes, increasing grazing distances, water harvesting and storage, and increased dependence on social welfare.	Dube and Jury (2000); Popoola et al. (2019); Gbetibouo et al. (2010); Hunter and Cronin (2020).
Conservation farming, store feeds, no crops planted, grey water, reducing herd size.	Fanadzo et al. (2021).
Purchase feed from the other farmers, resting the camp	Letsoalo et al. (2023).
Abandonment of livestock, change of grazing patterns, change of fodder.	Talanow et al. (2021).
Government aid, feed budgeting, reduce herd size, feed storage during summer, pasture management.	Lamega et al. (2021).
Diversification of crops and livestock.	Popoola et al. (2019); Zwane (2019); Maluleke et al. (2020); Lottering et al. (2021).
Adjusting the schedule of operations.	Maponya and Mpandeli (2013); Mare et al. (2018); Archer et al. (2021); Lottering et al. (2021); Meissner et al. (2014).
Adjusting stock routes and distances.	Dube and Jury (2000); Zwane (2019).
Introducing livestock farming systems that combine different methods, such as stall-feeding and pasture grazing.	Taruvinga et al. (2013); Meissner et al. (2014); Mapfumo et al. (2017); Mthembu and Zwane (2017); Popoola et al. (2019).
Alterations in livestock/herd composition, favoring the selection of larger animals over smaller ones.	Musemwa et al. (2012); Taruvinga et al. (2013); Meissner et al. (2014); Mthembu and Zwane (2017); Mapfumo et al. (2017).
Construction of shade to reduce heat	Popoola et al. (2019).
Ensuring more efficient nutrition, adjusting production systems to minimise their carbon footprint by reducing emissions, waste generation, and energy consumption, adapting production systems to reduce reliance on water for both production and irrigation. Adjusting production systems to decrease dependence on water for both production and irrigation.	Rust and Rust (2013).
Seasonal climate forecasting.	Mutengwa et al. (2023).
Diversification of feed resources and feeding strategies.	Molieleng et al. (2021).
Use of indigenous cattle breeds.	Mapiye et al. (2019).
Modification of land utilisation and water management for irrigation.	Mandleni and Anim (2011a); Musemwa et al. (2012); Taruvinga et al. (2013).

Complementary breeding of livestock is another significant strategy, focusing on improving desirable traits such as disease resistance, productivity, and adaptability to local environmental conditions (Dube and Jury 2000; Below et al. 2010; Mandleni and Anim 2011b; Rust and Rust 2013; Scholtz et al. 2013; Mare et al. 2018; Mpandeli et al. 2015; Nyoni et al. 2019; Popoola et al. 2019; Zwane 2019, Malusi et al. 2021; Halimani et al. 2021; Hawkins et al. 2022; Slayi et al. 2022). By selectively breeding animals with favorable characteristics, farmers can enhance the overall resilience of their herds and improve their ability to withstand environmental stressors (Doeschl-Wilson et al. 2021). Properly deriving resilience phenotypes based on specific breeding goals is crucial for the success of such breeding programs (Sánchez-Molano et al. 2020). As climate challenges become more pronounced, enhancing the performance resilience of farm animals through selective breeding can help mitigate the effects of adverse weather conditions and seasonal variations, contributing to the sustainability of livestock farming (Tsartsianidou et al. 2021).

Water resource management and environmental conservation are addressed through strategies like drilling boreholes, planting trees for shade, and constructing shade structures (Mpandeli et al. 2015; Katiyatiya et al. 2017; Mare et al. 2018; Nyoni et al. 2019; Pili 2020; Malusi et al. 2021; Zhou et al. 2022). These practices provide reliable water sources, create microclimates that benefit livestock well-being and mitigate heat stress, particularly during hot and dry periods. These practices not only provide reliable water sources but also create microclimates that benefit livestock well-being and help mitigate heat stress, especially during hot and dry periods (Ncongwane et al. 2021).

Drilling boreholes is a critical strategy for tapping into groundwater resources, helping to secure a continuous water supply particularly in arid and semi-arid regions where surface water is often insufficient or unpredictable. Boreholes provide a lifeline during droughts and periods of water scarcity, supporting agricultural activities, human consumption, and livestock hydration. Moreover, the strategic planting of trees for shade plays a crucial role in enhancing environmental conservation efforts. Trees act as natural water regulators by reducing evaporation rates from the soil and water bodies, thereby conserving water resources. The shade provided by trees also creates microclimates that are conducive to the well-being of livestock, offering respite from extreme heat and minimising heat stress-related health issues.

Financial management strategies, such as reducing production during challenging periods, seeking financial assistance and exploring migration as a temporary measure, are common practices to mitigate economic risks and ensure the sustainability of livestock operations (Below et al. 2010; Zwane 2019; Bahta 2020; Fanadzo 2021; Hunter and Cronin 2020; Bahta 2022; Bahta and Myeki 2021). Utilising savings, making strategic investments and accessing credit facilities contribute to long-term financial stability and operational resilience. Studies have shown that smallholder livestock farmers in South Africa require assistance from the government and stakeholders to enhance their resilience to food insecurity and minimise vulnerability (Myeki and Bahta 2021). Additionally, the implementation of sustainable extensification strategies, such as raising low-input livestock adapted to specific grazing conditions, is crucial for ensuring the financial sustainability of ranching operations (Holechek et al. 2020).

Selling livestock early is a key climate change adaptation and mitigation strategy used by smallholder farmers in South Africa to minimise losses during droughts and extreme weather conditions (Boone et al. 2004; Mandleni and Anim 2011b; Maluleke and Mokwena 2017; Bahta 2020). By reducing herd sizes in anticipation of feed and water shortages, farmers protect their remaining stock from malnutrition and mortality while securing immediate income to invest in alternative livelihood activities or restocking during favorable conditions. This strategy enhances resilience by preventing overgrazing, which can lead to land degradation and biodiversity loss, thereby contributing to sustainable land management.

Registering as livestock farmers is an important adaptation and mitigation strategy employed by smallholder farmers in South Africa to enhance resilience to climate change and improve access to institutional support (Maluleke and Mokwena 2017; Bahta 2022; Bahta and Myeki 2022). Formal registration allows farmers to benefit from government and private sector initiatives, including access to drought relief programs, veterinary services, training and financial support for climate-smart agriculture. This enhances their capacity to cope with climate variability by improving herd health, reducing mortality rates, and optimising production efficiency.

From a mitigation perspective, registration facilitates participation in sustainable grazing schemes promoting responsible livestock management and reducing greenhouse gas emissions. By securing formal recognition, smallholder farmers also strengthen their economic sustainability through better market access, improved value chain integration, and eligibility for financial services. Ultimately, registering as livestock farmers contributes to sustainability by fostering resilience, promoting environmentally sound livestock management, and ensuring long-term economic viability in the face of climate challenges.

Utilising savings and investments are vital adaptation and mitigation strategies for smallholder livestock farmers in South Africa, helping them manage climate risks and enhance resilience (Bahta 2022; Bahta and Myeki 2021). By saving income from livestock sales and investing in climate-smart practices such as improved water management systems, drought-resistant fodder, smallholder farmers can better withstand climate shocks like droughts and disease outbreaks. Savings provide a financial buffer, reducing reliance on emergency aid or distress sales during crises, while strategic investments in sustainable farming practices improve long-term productivity and environmental conservation. From a mitigation perspective, investing in efficient feeding systems and sustainable rangeland management helps reduce methane emissions and land degradation. These financial strategies contribute to sustainability by promoting economic stability, environmental resilience, and long-term food security, ensuring that smallholder farmers can continue livestock production while adapting to and mitigating climate change impacts.

Supplementation of livestock feed during forage scarcity ensures adequate nutrition and maintains productivity levels (Gbetibouo et al. 2010; Mandleni and Anim 2011b; Malusi et al. 2021; Lamega et al. 2021; Zhou et al. 2022; Letsoalo et al. 2023). Furthermore, some methods applied by smallholder to adapt and mitigate to climate change include alternative breeding objectives, such as raising drought-tolerant livestock breeds that are well-suited to local climatic conditions and this enhances resilience and reduces dependence on external inputs (Molieleng et al. 2021; Bahta 2022; Bahta and Myeki, 2023). Livestock breeds, such as Nguni cattle and indigenous goats, are naturally adapted to harsh conditions, requiring less water and feed while maintaining productivity, thus reducing vulnerability to climate-induced feed shortages. By improving herd efficiency and survival rates, farmers can sustain livelihoods and economic stability during drought periods. Additionally, drought-tolerant breeds contribute to mitigation efforts by reducing the overgrazing pressure on rangelands, promoting ecosystem balance, and lowering methane emissions per unit of production. This approach aligns with sustainability by ensuring long-term livestock productivity, conserving natural resources, and supporting climate-resilient food systems that secure livelihoods for smallholder farmers.

Innovative approaches like wildlife ranching are also being explored as alternative or complementary strategies to traditional livestock farming (Otieno et al. 2023). Wildlife ranching promotes biodiversity conservation, ecotourism, and diversified income streams, contributing to overall landscape sustainability (Otieno et al. 2023). However, challenges such as land tenure conflicts, poaching risks, and regulatory constraints need to be carefully managed to ensure the long-term viability of wildlife ranching. Overall, when implemented effectively, wildlife ranching presents a viable model for balancing economic development with environmental stewardship, contributing to a more sustainable and climate-resilient agricultural sector.

The use of crop residues is a viable and effective adaptation strategy to combat the impacts of climate change on agricultural systems (Lamega et al. 2021). By improving soil health, enhancing water retention, regulating temperature, and reducing greenhouse gas emissions, residues contribute to more resilient and sustainable farming practices. Changing the area of land grazed is another valuable adaptation strategy for mitigating the effects of climate change on livestock systems and pasture management (Dube and Jury 2000; Gbetibouo et al. 2010; Popoola et al. 2018; Hunter and Cronin 2020). This strategy, also known as adaptive or rotational grazing, involves adjusting grazing patterns to account for altered rainfall, temperature shifts, and ecosystem changes due to climate change.

Adaptation strategies like controlled feeding, livestock migration, quarantine, and buying feed are crucial for building resilience in livestock production systems in the face of climate change (Fanadzo 2021). These approaches help mitigate the impacts of extreme weather events, erratic rainfall, and shifting climatic conditions that affect fodder availability and animal health. Controlled feeding, strategic livestock migration, quarantine measures, and purchasing feed are part of adaptive management practices that optimise resource use, minimise disease risks, and improve farm efficiency (Fanadzo 2021).

Some other adaptation and mitigation strategies being utilised by smallholder livestock farmers include winter feeding (Goqwana et al. 2008), annual flush vegetation (Hahn et al. 2005), genetic diversity (Molotsi et al. 2017) and dipping and dosing (Mandleni and Anim, 2011). Continuous grazing under conservative stocking rates (Rasch et al. 2016; Malusi et al. 2021), wetting and forced ventilation (Ogundeji et al. 2021), seeking shade, extending limbs, wallowing in mud, and reducing food intake (Preez 2007) are additional strategies. The use of acaricides every fortnight and considering hair length and coat color when acquiring cattle (Katiyatiya et al. 2014), increasing grazing distances, changing grazing routes, and water harvesting and storage (Popoola et al. 2019) are other methods. Furthermore, conservation farming (Fanadzo et al. 2021), and in extreme cases, the abandonment of livestock (Talanow et al. 2021) are part of these strategies. When implemented collectively, these strategies contribute to the sustainability of smallholder livestock systems by promoting environmental stewardship, economic viability, and social resilience in the face of climate change.

Adaptation measures such as government support, feed budgeting, reducing herd size, storing feed during summer and pasture management (Lamega et al. 2021) are critical strategies for ensuring the sustainability of agricultural systems in the face of climate change. These measures enhance resource efficiency, minimize environmental degradation, and promote long-term agricultural viability. For instance, effective pasture management prevents overgrazing, maintains soil health, and conserves biodiversity, while feed storage and budgeting optimise resource use, reducing reliance on external inputs. Additionally, diversification of crops and livestock (Zwane 2019; Popoola et al. 2019; Maluleke et al. 2020) strengthens resilience by spreading risk across multiple enterprises, ensuring food security and improving farmers’ economic stability.

Diversified systems are less vulnerable to climate shocks, pests and diseases, making them more sustainable in the long run. Furthermore, government support in the form of policy incentives, financial aid and extension services plays a crucial role in enabling farmers to adopt sustainable practices that align with climate adaptation and ecological preservation. Collectively, these strategies contribute to sustainability by fostering resilient agricultural systems that balance productivity, environmental conservation, and economic well-being.

Adjusting the schedule of operations (Maponya and Mpandeli 2013; Mare et al. 2018; Archer et al. 2021; Lottering et al. 2021) and introducing livestock farming systems that combine different methods, such as stall-feeding and pasture grazing (Taruvinga et al. 2013; Meissner et al. 2014; Mthembu and Zwane 2017 Mapfumo et al. 2017), are key strategies. Altering livestock/herd composition, favoring the selection of larger animals over smaller ones (Musemwa et al. 2012; Taruvinga et al. 2013; Meissner et al. 2014; Mapfumo et al. 2017; Mthembu and Zwane 2017) and constructing shade to reduce heat (Popoola et al. 2019) are additional adaptation methods that are being applied by smallholder livestock farmers.

According to Rust and Rust (2013), effective mitigation and adaptation strategies involve enhancing efficiency in nutrition, optimising production systems to minimise their carbon footprint by reducing emissions, waste generation, and energy consumption. Adaptation measures include modifying production systems to lessen reliance on water for both production and irrigation, which is critical in water-scarce environments. Additionally, breeding and cultivating drought-tolerant dryland crops and pastures for dairy feed play a crucial role in sustaining livestock production under changing climatic conditions. Implementing more efficient and eco-friendly agricultural practices further contributes to sustainability by improving resource use efficiency while reducing environmental degradation. These strategies collectively support resilience against climate change while promoting sustainable agricultural development.

Seasonal climate forecasting and effective early warning systems play a critical role in enhancing the resilience of livestock farmers to climate change (Mutengwa et al. 2023). By providing timely and accurate predictions of weather patterns, including droughts, heatwaves, and extreme rainfall events, seasonal climate forecasting allows farmers to make proactive decisions regarding grazing management, water conservation and feed planning. Early warning systems further strengthen adaptive capacity by alerting farmers and other stakeholders to imminent climate-related disasters, enabling them to implement precautionary measures such as destocking, securing alternative feed sources and adjusting herd management strategies. These interventions help minimise livestock losses, reduce economic shocks and sustain livelihoods, ultimately contributing to climate-smart livestock production and long-term agricultural sustainability.

The diversification of feed resources and feeding strategies is a crucial mitigation and adaptation strategy for enhancing the resilience of livestock systems to climate change (Molieleng et al. 2021). By incorporating alternative and locally available feed resources, such as drought-tolerant forages and nutrient-rich crop residues, smallholder farmers can reduce reliance on conventional feed sources that may become scarce or expensive due to climate variability. These approaches not only enhance feed availability and nutritional quality but also contribute to reducing greenhouse gas emissions from livestock production, promoting sustainable and climate-resilient animal agriculture.

Indigenous cattle breeds serve as a crucial adaptation and mitigation strategy for climate change among smallholder livestock farmers due to their resilience to harsh environmental conditions, low input requirements, and ability to utilise marginal rangelands (Mapiye et al. 2019). These breeds, such as the Nguni and Tuli exhibit superior adaptability to heat stress, drought tolerance, and resistance to endemic diseases, reducing the need for veterinary interventions and supplementary feeding. Their efficient feed conversion and ability to graze on diverse forages enhance resource use efficiency, contributing to sustainable livestock production in changing climates.

According to Mandleni and Anim (2011c), Musemwa et al. (2012), and Taruvinga et al. (2013), some adaptation and mitigation strategies include modification of land utilisation and water management for irrigation. These strategies enhance sustainability by promoting efficient resource use, reducing environmental degradation, and improving resilience to climate change. Modifying land utilisation through rotational grazing, pasture rehabilitation, and agroforestry helps prevent soil erosion, maintain biodiversity, and support long-term productivity. Similarly, sustainable water management practices such as rainwater harvesting, conservation agriculture, and the use of drought-resistant fodder crops ensure reliable water availability for livestock and crop production. By integrating these strategies, smallholder farmers can sustain their livelihoods while minimizing their ecological footprint, contributing to climate resilience, food security, and the overall sustainability of agricultural systems.

These adaptation and mitigation strategies collectively contribute to building more resilient and sustainable livestock production systems in South Africa, preparing farmers to face future challenges and uncertainties. Collaboration among farmers, researchers, policymakers, and extension services is essential for promoting the adoption of these strategies and ensuring the long-term sustainability of the livestock sector in the region.

### Factors influencing the adoption of adaptation and mitigation strategies

Factors influencing the adoption of adaptation and mitigation strategies in livestock production in South Africa encompass a range of demographic, environmental, socioeconomic, and knowledge-related themes (See Table 2). Understanding these factors is crucial for promoting the widespread uptake of sustainable practices and enhancing the resilience of the livestock sector.

**Table 2:** Factors influencing the adoption of adaptation and mitigation strategies

Classification	Themes	Authors
Demographic	Gender. Marital status. Age of farmers and Landowners.	Mandleni and Anim (2011a). Mandleni and Anim (2011b). Oduniyi et al. (2020a); Tshikolomo et al. (2022).
	Education	Slayi et al. (2023b).
Environmental	Temperatures. Lack of access to water. Increase in temperature. Ecosystem disservices with farmers avoiding poisonous plants and boggy areas.	Mandleni and Anim (2011). Gbetibouo et al. (2010). Otieno et al. (2023). O’Farrell et al. (2007).
Socio-economic	Health Status. Use of labor, other sources of income. Level of education. Access to formal extension services. Employment Status. Lack of access to credit. Community involvement and social networks Farm experience, income, land ownership, government assistance, cooperatives. Limited farming resources, short-term planning and lack of finances, and perception on climate change. Cultural and traditional practices.	Tshikolomo et al. (2022). Oduniyi et al. (2020b). Mandleni and Anim (2011). Bahta (2020); Tshikolomo et al. (2022). Ojo et al. (2021). Gbetibouo et al. (2010). Popoola et al. (2019); Bahta (2020). Pili (2022); Zhou et al. (2022); Hawkins et al. (2022). Mubecua et al. (2024).
Knowledge-related	Lack of drought awareness. Access to weather information. Training. Limited knowledge about drought impacts and adaptation strategies. Information received about climate change, Formal extension.	Pili (2020); Pili (2022). Bahta (2020); Mdiya et al. (2024). Mandleni and Anim (2011). Bahta (2020); Pili (2022). Mandleni and Anim (2011b).
Networks	Social networks and community influence.	Popoola et al. (2019); Bahta and Myeki (2022).
Resources	Availability and quality of resources, such as pasture and water	Zuma-Netshiukwi (2023).
Perception and awareness.	Awareness of benefits and challenges.	Slayi et al. (2023b).
Credit access	Access to credit and financial services.	Ojo et al. (2021).

Demographic factors play a significant role in shaping farmers’ decisions regarding adaptation and mitigation strategies (Mandleni and Anim 2011a; Oduniyi et al. 2020a; Tshilomo et al. 2022; Slayi et al. 2023b). Gender dynamics, marital status, and the age of farmers and landowners influence their perspectives, priorities, and capabilities in implementing sustainable practices. For instance, younger farmers may be more open to adopting innovative technologies, while older farmers with more experience may rely on traditional knowledge but could be resistant to change. Higher levels of education and knowledge about the benefits and challenges of adaptation strategies, such as communally established feedlots, positively influence farmers willingness to adopt these strategies (Slayi et al. 2023b). Ignoring these demographic nuances risks oversimplifying their impact and overlooks how intersectional factors such as age, gender, and education interact to either enable or hinder the adoption of sustainable practices across different farmer populations.

Environmental factors such as temperatures, water availability, and climatic changes directly impact the feasibility and effectiveness of adaptation strategies (O’Farrell et al. 2007; Gbetibouo et al. 2010, Mandleni and Anim, 2011; Otieno et al. 2023). High temperatures and water scarcity, exacerbated by climate change, pose challenges to livestock farming and necessitate adaptive measures. Farmers in regions facing environmental stress often focus on strategies such as water conservation, managing heat stress, and utilizing drought-resistant breeds. However, a more in-depth analysis of how these strategies are adopted may be necessary. This could include factors such as access to resources, socio-economic constraints, knowledge dissemination, and policy support, all of which can significantly influence the successful implementation of sustainable practices in livestock production systems. Additionally, cultural preferences, risk perceptions, and the local ecological context may further shape how and to what extent these strategies are adopted. Without addressing these multidimensional factors, the broader adoption of sustainable livestock practices may face substantial barriers.

Socioeconomic factors significantly influence farmers’ capacity to adopt adaptation and mitigation strategies (Gbetibouo et al. 2010, Mandleni and Anim, 2011; Bahta 2020, Oduniyi et al. 2020b; Pili, 2022; Tshikolomo et al. 2022; Zhou et al. 2022; Hawkins et al. 2022; Mubecua et al. 2024). Health status, access to labor and alternative income sources, educational level, formal extension services, employment status, credit accessibility, community engagement, and resources like land ownership and government assistance all play crucial roles. Limited resources, financial constraints, and short-term planning can hinder the adoption of sustainable practices, highlighting the need for supportive policies, financial incentives, and capacity-building programs.

Knowledge-related factors, including awareness of drought risks, access to weather information, training opportunities, and understanding of adaptation strategies, influence farmers’ readiness to implement sustainable practices (Mandleni and Anim, 2011; Mandleni and Anim 2011b; Bahta 2020; Pili 2020; Pili, 2022). While these factors highlight the importance of knowledge in shaping adaptation efforts, a more detailed exploration of strategy adoption may be lacking. Crucial aspects such as the quality, timeliness, and relevance of information, as well as the role of local knowledge systems and networks, are often overlooked. Moreover, factors like trust in institutions, peer influence, and policy incentives play a significant role in shaping decision-making. A comprehensive understanding of these interconnected elements is essential for enhancing the implementation of sustainable livestock production systems.

The influence of social networks and community interactions also plays a role (Popoola et al. 2019; Bahta and Myeki 2022). Farmers who are part of supportive communities and have access to peer learning and sharing experiences are more likely to adopt adaptation strategies. However, the depth of these interactions is often understated. Trust within networks, the frequency of interactions, and the structure of community ties whether formal or informal can significantly impact the effectiveness of knowledge transfer and the adoption of sustainable practices. Moreover, the role of local opinion leaders and influencers within these communities may be pivotal in setting examples for others, driving collective action, and ensuring long-term commitment to adaptive strategies. A more nuanced exploration would also consider barriers such as unequal access to social networks and how marginalized groups, particularly women and small-scale farmers, may face limitations in benefiting from these community resources, potentially hindering the widespread implementation of sustainable livestock practices.

Quality and availability of resources, such as pasture and water, influence the effectiveness and feasibility of adaptation strategies (Zuma-Netshiukwi 2023). Variability in resource availability due to seasonal changes, climate variability, or geographic disparities can significantly affect the capacity of farmers to implement sustainable practices. For instance, during droughts or prolonged dry periods, limited access to quality pasture or water may hinder the adoption of strategies like rotational grazing or water-efficient technologies. Furthermore, the quality of these resources directly impacts livestock health and productivity, making it vital to assess the alignment between resource availability and the specific adaptation strategies being considered. Ignoring these dynamics could lead to a one-size-fits-all approach that overlooks the unique environmental conditions farmers face, particularly in resource-scarce areas. Therefore, a comprehensive exploration must consider how resource constraints or abundance influence the practicality, timing, and success of sustainable practices in various contexts.

Farmers’ awareness of the benefits and challenges associated with adaptation strategies, such as increased livestock productivity and initial investment costs, affects their willingness to adopt these strategies (Slayi et al. 2023b). Farmers with a clear understanding of the long-term economic benefits, such as improved herd health and resilience to climate variability, may be more inclined to invest in sustainable practices, even if upfront costs are high. However, the perception of risk, lack of financial literacy, and uncertainties regarding the return on investment can deter adoption. Additionally, farmers’ ability to weigh short-term costs against long-term gains is influenced by their socioeconomic status and access to financial resources, which can lead to unequal adoption rates. Ignoring these nuances risks overlooking key drivers of decision-making, particularly among resource-constrained farmers, who may perceive the costs of adaptation as prohibitive, despite its potential benefits for sustainability in livestock production systems.

Access to credit and financial services enables farmers to invest in adaptation and mitigation strategies. Farmers with access to credit tend to be more inclined to implement these practices (Kumari et al. 2019; Ojo et al. 2021). Access to financial resources can lower the barrier to adopting capital-intensive practices, such as improved feed systems, water conservation technologies, or rotational grazing infrastructure. Additionally, farmers’ financial literacy and their trust in financial institutions influence how effectively they can leverage credit to implement sustainable practices. Access to credit is also often unequal, with smallholder farmers, women, and marginalized groups frequently facing more significant challenges in obtaining financial services due to systemic barriers or lack of collateral. This unequal access may prevent some farmers from benefiting from available adaptation opportunities, thus exacerbating disparities in the implementation of sustainable practices across different regions and farming communities. Ignoring these factors risks oversimplifying the impact of credit on strategy adoption and may overlook the need for tailored financial services to support more inclusive adoption of sustainable livestock production systems.

Increasing awareness, providing relevant information and training, and promoting knowledge-sharing platforms are essential for enhancing farmers’ capacity to cope with climate-related challenges and adopt resilient farming practices (Slayi et al. 2023b). Moreover, there is a pressing need for knowledge-sharing platforms and training programs aimed at increasing awareness and understanding of these strategies among farmers, extension services, researchers, and policymakers (Idrissou et al. 2023). This includes promoting the adoption of innovative technologies, supporting indigenous knowledge and traditional practices, and investing in research to evaluate the effectiveness of different strategies in the local context (Abdeta, 2020). Addressing these diverse factors requires a holistic approach that integrates demographic, environmental, socioeconomic, and knowledge-related considerations into policy frameworks, extension services, capacity-building initiatives, and community engagement efforts. Collaboration should be fostered between stakeholders from various fields such as agriculture, environmental science, and economics, to enrich the discussion on sustainable livestock production practices.

## Conclusion and recommendation

The study revealed the critical role of mitigation and adaptation strategies in ensuring the sustainability of livestock production, using South Africa as a case study amidst climate change. The findings reveal a diverse array of strategies adopted by farmers, including drilling boreholes, wildlife ranching, crop residues, changing area of land grazed, complementary breeding, water resource management, tree planting, feed supplementation, selling livestock early, and controlled feeding practices. To further enhance the resilience and long-term viability of the livestock sector, it is imperative to integrate these strategies into comprehensive policy frameworks and capacity-building initiatives. Policymakers should prioritise the integration of adaptation and mitigation measures into agricultural policies, providing economic incentives such as financial assistance to encourage sustainable practices among farmers. To enhance the resilience and long-term sustainability of the livestock sector, it is imperative to integrate these strategies into comprehensive policy frameworks and targeted capacity-building initiatives. Importantly, such frameworks must be underpinned by inclusive public policies that explicitly address and reduce gender, age, and access-related disparities particularly in access to land, water, credit, extension services, and markets.

Moreover, there is a pressing need for knowledge-sharing platforms and training programs aimed at increasing awareness and understanding of these strategies among farmers, extension services, researchers, and policymakers. This includes promoting the adoption of innovative technologies, supporting indigenous knowledge and traditional practices, and investing in research to evaluate the effectiveness of different strategies in the local context. Collaborative partnerships among stakeholders including those in agriculture, environmental science and economics are essential for co-designing and implementing strategies tailored to the specific challenges faced by South African livestock producers. To move beyond general calls for integration, concrete mechanisms should be established to facilitate effective collaboration. These could include technical platforms for data sharing and innovation exchange, living labs where experimental practices are tested on-farm with active farmer participation, and participatory committees that bring together local farmers, technicians, economists and researchers to co-develop solutions. Supporting these initiatives through capacity building, inclusive research programs, and the respectful integration of indigenous knowledge with modern practices will enhance the resilience and sustainability of South Africa’s livestock sector. Such a framework can contribute meaningfully to food security, lower greenhouse gas emissions, and improved adaptation to climate change. Future studies could benefit from using agro-ecological frameworks where such data are available.

### Study limitations

One limitation of this study is its focus on the provincial boundaries of South Africa as the primary geographic categorisation. While this approach aligns with how the majority of included studies reported their findings, it may not fully capture the ecological variability that influences adaptation and mitigation strategies in livestock production. Future studies could benefit from adopting agro-ecological frameworks, where such data are available, to allow for more ecologically meaningful comparisons and a deeper understanding of context-specific practices and challenges.

## Data Availability

The datasets used in this study can be obtained from the corresponding author upon request.
